# Evaluation of Immune and Vaccine Competence in Steroid-Sensitive Nephrotic Syndrome Pediatric Patients

**DOI:** 10.3389/fimmu.2021.602826

**Published:** 2021-03-12

**Authors:** Manuela Colucci, Eva Piano Mortari, Federica Zotta, Francesco Corrente, Carlo Concato, Rita Carsetti, Francesco Emma, Marina Vivarelli

**Affiliations:** ^1^Renal Diseases Research Unit, Genetics and Rare Diseases Research Area, Bambino Gesù Children's Hospital, IRCCS, Rome, Italy; ^2^Diagnostic Immunology Research Unit, Multimodal Medicine Research Area, Bambino Gesù Children's Hospital, IRCCS, Rome, Italy; ^3^Division of Nephrology, Department of Pediatric Subspecialties, Bambino Gesù Children's Hospital, IRCCS, Rome, Italy; ^4^Diagnostic Immunology Unit, Department of Laboratories, Bambino Gesù Children's Hospital, IRCCS, Rome, Italy; ^5^Division of Virology, Department of Laboratories, Bambino Gesù Children's Hospital, IRCCS, Rome, Italy

**Keywords:** vaccine competence, IgG, pediatric nephrology, steroid-sensitive nephrotic syndrome, immune competence, ELISPOT

## Abstract

Idiopathic nephrotic syndrome is a childhood renal disease characterized by a damage of the glomerular filtration barrier leading to an intense leakage of proteins into the urine. This severe proteinuria causes a transient but strong reduction of serum IgG. Therefore, evaluation of vaccine competence by measuring serum levels of protective antibodies can be misleading in nephrotic syndrome, especially during the active phase of disease. To overcome this issue, in parallel to measuring serum antigen-specific IgG, we quantified by ELISPOT the number of antigen-specific memory B cells induced by previous immunization with tetanus and hepatitis B virus (HBV) in 11 steroid-sensitive nephrotic syndrome (SSNS) pediatric patients at onset before any immunosuppressive treatment (mean age 5.1±0.9 years). Five age-matched children with non-immunomediated nephro-urologic disorders were also enrolled as controls (mean age 6.9±2.3 years). Low total serum IgG levels (<520 mg/dl) were found in all the analyzed SSNS patients. In parallel, median levels of anti-tetanus and anti-HBV IgG were significantly reduced compared to controls [0.05 (0.03–0.16) vs. 0.45 (0.29–3.10) IU/ml and 0.0 (0.0–0.5) vs. 30.3 (5.5–400.8) mIU/ml, respectively; *p* = 0.02 for both], with serum IgG titers below protective threshold in 7/11 SSNS patients for tetanus and in 9/11 SSNS patients for HBV. In contrast, all SSNS patients had a competent B-cell response, showing an amount of total IgG-secreting B cells >1,000 counts/10^6^ stimulated cells. The amount of anti-tetanus and anti-HBV IgG-secreting B cells was also comparable to that of controls (*p* = 0.24, *p* = 0.32, respectively), with a frequency of memory anti-tetanus and anti-HBV IgG secreting B cells >0.1% of total IgG secreting B cells. In conclusion, SSNS children at disease onset pre-immunosuppressive therapy showed a competent immune and vaccine response against tetanus and HBV, which can be correctly evaluated by quantification of antigen-specific memory B cells rather than by measuring serum IgG levels. This approach allows early identification of the impairment of immune and vaccine competence, which may derive from protracted use of different immunosuppressive drugs during disease course.

## Introduction

Idiopathic nephrotic syndrome (INS) is the most frequent glomerular disease in childhood. However, it has a rare incidence (1–17 cases per 100.000 children per year) (1). INS is characterized by a damage of the glomerular permeability barrier, which causes a severe leakage of proteins into the urine, associated with hypoalbuminemia and edema (1). A strong reduction of serum IgG associated with increased serum IgM levels is also frequent during the active phase of disease and sometimes persists also during remission (2, 3). Whether it depends on an impairment of the immune homeostasis or just on the intense proteinuria is debated (2–6). Several T-cell dysregulations have indeed been described both in relapse and in remission (7, 8) and altered levels of memory B cells have been observed already at disease onset, before any immunosuppressive therapy (9). The reduction of protective antibodies observed in INS patients can also be dependent on the prolonged and intense immunosuppression administered in severe forms of the disease, increasing the risk for these patients to develop severe infections (10, 11). At disease onset, patients are treated with a standardized protocol of oral prednisone therapy, to which most patients respond within 4–6 weeks (defined as “steroid-sensitive nephrotic syndrome” patients, SSNS). Within the majority of pediatric patients affected by SSNS, clinical evolution can be extremely heterogeneous, ranging from non-relapsing to severely steroid-dependent forms, which require repeated cycles of steroid therapy and further immunosuppression with one or more steroid-sparing drugs, including anti-proliferative agents, calcineurin inhibitors and B-cell depleting drugs (1). This intense and prolonged immunosuppression can strongly impact immune and vaccine competence in severe forms of SSNS (10, 11).Whether this competence of SSNS pediatric patients is impaired only by the intense and prolonged immunosuppression required to maintain the disease remission or whether the intrinsic immune dysregulation can contribute to this impairment is not clear. Whatever the mechanism behind the lowering of serum IgG titers, this reduction hampers the correct evaluation of the immune and vaccine competence which is usually based on the dosage of total and antigen-specific serum IgG titers.

The aim of this pilot observational study is to evaluate the immune and vaccine competence of SSNS pediatric patients at disease onset, prior to any immunosuppressive treatment. To this purpose, we quantified antigen-specific memory B cells in parallel to the dosage of serum protective IgG.

## Materials and Methods

### Study Patients

This monocentric observational study was conducted among INS pediatric patients followed from July 2018 to June 2020 at the Bambino Gesù Children's Hospital, IRCCS in Rome, Italy. The study was approved by our Ethics Committee and was conducted in compliance with the declaration of Helsinki. Written informed consent on behalf of the minors/children enrolled was obtained from parents. All patients at disease onset accessing our clinic in the study period and consenting to participate in the study were enrolled before starting oral prednisone therapy at a standard protocol of 60 mg/m^2^/daily for 6 weeks followed by 40 mg/m^2^/every other day for 6 weeks. Patients were then monitored for the response to prednisone therapy and defined “steroid-sensitive” (SSNS) if they responded within 4 weeks by showing negative proteinuria on urine dipstick for ≥ 3 days (12). Patients who did not respond to the standardized prednisone therapy within 4 weeks [defined as “steroid-resistant” (12)] were subsequently excluded. Excluding criteria were also chronic infections, previous treatment with immunosuppressive drugs (excluding low dose steroids for periods <3 months), age >18 years. Renal biopsy was considered only for patients ≤ 1 or ≥12 years old, sustained elevation of serum creatinine or findings indicative of another immune-mediated disorder (1). Age-matched non immune-mediated nephro-urologic disorders were also evaluated as controls (CTRL). Clinical and demographical characteristics were registered.

### Cell Collection

An additional blood sample to perform the evaluation of immune and vaccine competence was obtained at the first hospital admission for SSNS patients and during routine visits for CTRL. Peripheral blood mononuclear cells (PBMCs) were isolated by Ficoll-Paque Plus (Amersham Biosciences) density-gradient centrifugation and cryopreserved in liquid nitrogen up to analysis.

### CpG Stimulation and ELISPOT

This assay was performed on previously anonymized samples in a blinded fashion in order to minimize a potential bias of data analysis. Immune and vaccine competence were determined by evaluating the ability of stimulated B cells to produce total and antigen-specific immunoglobulins, respectively.

PBMCs were cultured in complete medium at a concentration of 1 × 10^6^ cells/ml. Complete medium was prepared as follows: RPMI-1640 (Euroclone), 10% heat inactivated fetal bovine serum (FBS, Hyclone Laboratories), 1% L-Glutammine (GIBCO BRL); 1% Penicillin/Streptomicin 100 × (Euroclone), 1% sodium pyruvate (GIBCO BRL).

Cells were stimulated for 5 days with 0.35 μM TLR9 agonist CpG-B ODN2006 (Hycult Biotech) plus 20 ng/ml rhIL-21 (Milteny) and 20 ng/ml rhIL-4 (Milteny).

For the simultaneous detection of IgM and IgG we used the Human IgG/IgM Dual-Color B Cell ELISpot Kit (R&D System). This kit is designed for the simultaneous detection of total and antigen specific IgM and IgG.

For the detection of total IgG and IgM polyclonal antibodies specific for human IgG and IgM, respectively, were coated onto a polyvinylidene difluoride (PVDF)-backed microplate following manufacturer's instructions. For the detection of antigen specific memory B cells microplate were coated overnight with recombinant hepatitis B surface Ag (HbsAg adw), (Prospec) and with synthetic tetanus toxin peptide (C-term), (OriGene).

PBMCs stimulated for 5 days, as described before, were collected, counted and seeded in the coated plates. Plates were left at 37°C, 2% CO_2_ for overnight to allow antibody secretion. A total of three 1:2 serial dilutions were done starting in the first well with 2 × 10^4^ cells for detection of total IgG and IgM. A total of 2 × 10^5^ cells were seeded in the first dilution well (three 1:2 serial dilutions) for the detection of B cells secreting specific antibodies.

After washing, a horseradish peroxidase-conjugated polyclonal antibody specific for IgG and a biotinylated polyclonal antibody specific for human IgM were added to the wells. Following a wash, alkaline-phosphatase conjugated to streptavidin was added and a substrate solution (BCIP/NBT) was added. After washing the BCIP/NBT from the wells with deionized water, an AEC chromogen solution was added to the wells. A red precipitate and a blue-black colored precipitate formed and appeared as spots, with each red spot representing an individual IgG secreting cell and each blue spot representing an individual IgM secreting cell. Plates were left to dry before counting with an ELISCAN (A-EL-VIS).

### Laboratory Analytes

Hematology (serum protein, serum albumin, serum creatinine, C reactive protein) and urinary (protein-to-creatinine ratio) parameters and serum IgG, IgA, IgM, anti-tetanus IgG and anti- hepatitis B virus (HBV) IgG were measured as routine analysis. Normal ranges for serum IgG (520–1,500 mg/dl), IgA (36–320 mg/dl) and IgM (35–155 mg/dl) as well as antibody titers determining sufficient (protective) immunization against HBV (>10 mIU/ml) and tetanus (>0.6 IU/ml) were indicated in the diagnostic laboratory of Bambino Gesù Children's Hospital – IRCCS. Range for antibody titers representing an existing (but not sufficient) immunization against tetanus (0.1–0.6 IU/ml) was also reported (13).

### Statistical Analyses

This is a single center, pilot study. As there is no null hypothesis to test, no formal sample size calculation was performed. Continuous data were expressed as mean ± standard error of the mean (SEM) if they passed normality test (Shapiro-Wilk test), or medians and interquartile range otherwise; categorical data were represented as numbers and percentages. Differences between groups were compared by unpaired *t*-test for normally distributed data or by Mann-Whitney U test for non-parametric data; Fisher exact test was used to compare proportions of patients in different categorical variables. *P*-values < 0.05 were considered significant. Analyses were performed through the software GraphPad Prism 6.

## Results

### Patient Characteristics

Twelve INS patients (five males and seven females) at disease onset were enrolled for the current study. One patient who did not respond to steroid treatment within 4 weeks was subsequently excluded from the analysis, which was performed on the remaining 11 SSNS patients. Five age-matched controls (three males and two females) with non-immune-mediated nephro-urologic disorders (one chronic kidney disease, one kidney stone, one kidney hypodysplasia, two nephrocalcinosis with hypercalcemia) were also enrolled. Mean time to remission of SSNS patients was 8.0±0.6 days from starting prednisone treatment. Table 1 summarizes demographical and clinical characteristics. Mean age was 5.1±0.9 years for SSNS patients and 6.9±2.3 years for CTRL. Only one SSNS patient underwent renal biopsy since he was ≥12 years old at onset and presented a histological pattern suggestive of minimal change disease. As expected, serum protein and serum albumin were significantly lower and proteinuria was significantly higher in SSNS patients compared to CTRL (Table 1). Serum levels of C-reactive protein were normal (<0.5 mg/dl) in all patients.

**Table 1:** Characteristics of study patients.

**Parameter**	**Unit**	**SSNS at onset (*n* = 11)**	**CTRL (*n* = 5)**	***P*-value**
**Demographics**				
Age	Years	5.1 ± 0.9	6.9 ± 2.3	0.38
Male sex	*N* (%)	5 (45)	3 (60)	1.0
**Clinical characteristics**				
Serum Protein	g/dl	4.1 ± 0.1^*^	6.9 ± 0.2	**<0.001**
Serum albumin	g/dl	2.2 ± 0.1^*^	4.7 ± 0.1	**<0.001**
Serum Creatinine	mg/dl	0.3 [0.2–0–4]	0.4 [0.3–1.8]	0.11
C reactive protein >0.5 mg/dl	*N* (%)	0 (0)	0 (0)	**-**
Urinary protein-to-creatinine ratio	mg/mg	18.3 ± 4.1	0.2 ± 0.1	**0.01**
Time to remission	Days	8.0 ± 0.6	-	-

Continuous data are expressed as mean ± standard error of the mean or median [interquartile range] and compared by unpaired t test or Mann-Whitney U test, respectively. Categorical values are indicated as absolute count and percentage, compared by a Fisher's exact test. SSNS, steroid-sensitive nephrotic syndrome; CTRL, control group.

* *All SSNS patients had already received albumin infusions at time of sampling. The bold numbers highlight the significant differences*.

### Serum Immunoglobulin Levels and Immune and Vaccine Competence

**Figure 1:**
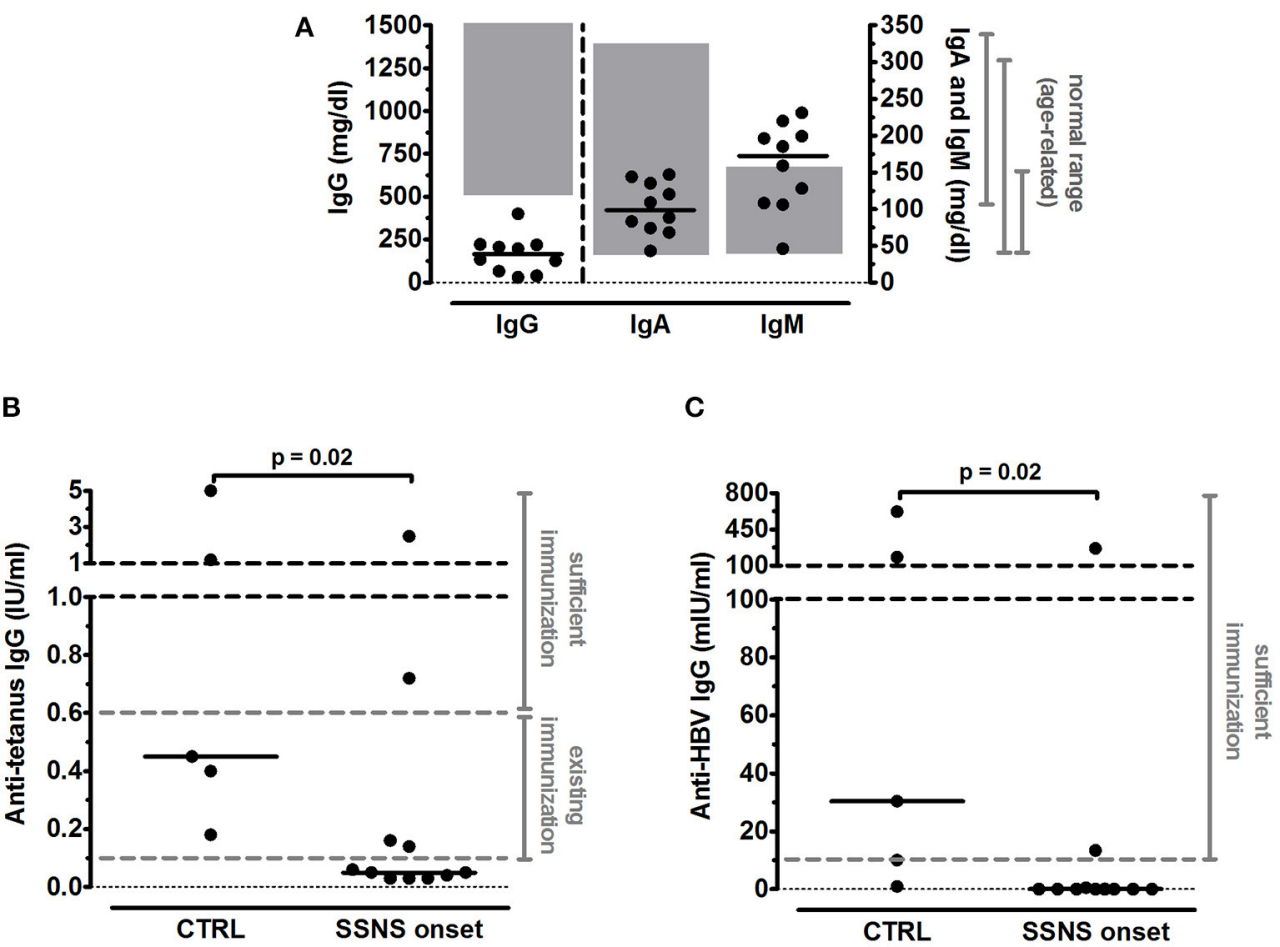
Serum immunoglobulin levels in steroid-sensitive nephrotic syndrome pediatric patients at onset. **(A)** Levels of total serum IgG, IgA and IgM were measured in steroid-sensitive nephrotic syndrome pediatric patients at disease onset (SSNS, *n*=10/11) and expressed as mg/dl. In one patient serum immunoglobulin levels were not determined. Each plot represents a different patient. Gray areas represent the age-related normal range as indicated by the diagnostic laboratory of Bambino Gesù Children's Hospital, IRCCS. **(B,C)** Antigen-specific IgG titers against **(B)** tetanus and **(C)** hepatitis B virus (HBV) were measured in SSNS pediatric patients at onset (*n*=11) and in age-matched controls (CTRL, *n*=5) and expressed as IU/ml and mIU/ml, respectively. Protective levels identified by dashed gray lines were indicated in the diagnostic laboratory of Bambino Gesù Children's Hospital, IRCCS. Horizontal lines indicate the medians and differences between groups were compared using the Mann–Whitney *U* test.

All patients were previously vaccinated against tetanus and HBV as per national requirements (14): in the first year of age, all children received three doses of both tetanus and HBV vaccines; a fourth booster dose of tetanus vaccine was administered in 4 SSNS patients and in 2 CTRL who were older than 6 years. Mean time elapsed since last immunization was not significantly different between the two groups both for tetanus (2.5±0.5 years for SSNS patients vs. 4.2±1.2 years for CTRL, *p* = 0.13) and HBV (4.1±0.9 years for SSNS patients vs. 6.0±2.3 for CTRL, *p* = 0.38). Serum IgG were below the age-related normal range (520–1,500 mg/dl) in 10/10 analyzed SSNS patients (mean levels = 164.3±35.0 mg/dl) and serum IgM were higher than age-related normal range (35–155 mg/dl) in 6/10 SSNS patients (mean levels = 157.8±18.8 mg/dl) (Figure 1A). In contrast, no alteration was observed in serum IgA levels (mean levels = 101.1±11.2 mg/dl) (Figure 1A).

Serum anti-tetanus IgG titers were below the level of sufficient protection (0.6 IU/ml) in 9/11 SSNS patients and below the existing protection (0.1 IU/ml) in 7/11 SSNS patients, respectively (Figure 1B) and median levels were significantly reduced in SSNS patients compared to CTRL (0.05 [0.03–0.16] vs. 0.45 [0.29–3.10] IU/ml, *p* = 0.02; Figure 1B). In parallel, serum anti-HBV IgG titers were undetectable in 9/11 SSNS patients (Figure 1C) and were significantly lower in SSNS patients compared to CTRL [0.0 (0.0–0.5) vs. 30.3 (5.5–400.8) mIU/ml, *p* = 0.02, Figure 1C].

**Figure 2:**
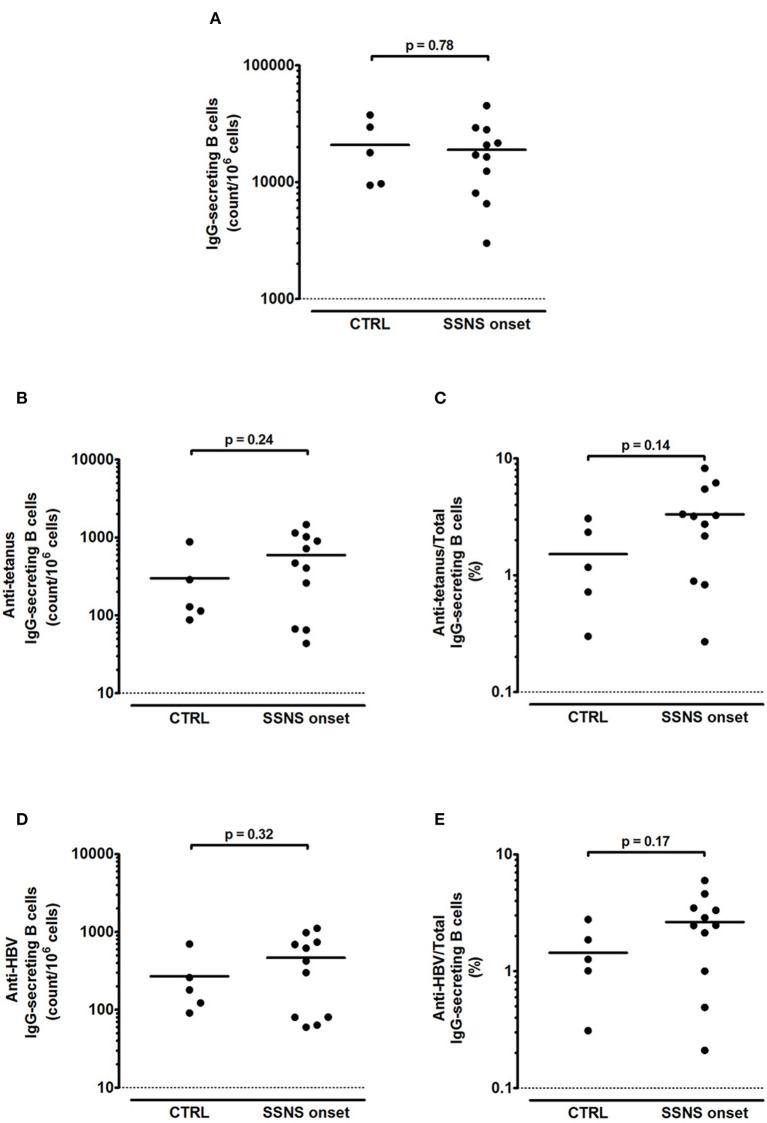
Total and antigen-specific IgG-secreting B cells in steroid-sensitive nephrotic syndrome pediatric patients at onset. **(A–E)** Isolated PBMCs were stimulated for 5 days with CpG plus rhIL-21 and rhIL-4. Following stimulation, **(A)** total, **(B,C)** anti-tetanus and **(D,E)** anti-HBV IgG-secreting B cells were enumerated by ELISPOT in steroid-sensitive nephrotic syndrome pediatric patients at disease onset (SSNS, *n*=11) and in age-matched controls (CTRL, *n*=5). Antigen-specific memory B cells were represented as **(B,D)** absolute count/10^6^ cells and as **(C,E)** percentage of total IgG-secreting B cells. Each plot represents a different patient. Horizontal lines indicate the means and differences between groups were compared using the unpaired *t* test.

In contrast to the reduced levels of serum IgG, SSNS patients showed an intact B-cell memory pool as demonstrated by the competent immune response to polyclonal stimulation (Figure 2A). The amount of total IgG-secreting B cells was > 1,000 counts/10^6^ stimulated cells in all SSNS patients and, despite interpersonal variability, no significant difference was observed as compared to CTRL (*p* = 0.78, Figure 2A). In parallel, also a competent memory B-cell response against tetanus and HBV was observed in SSNS patients compared to CTRL (*p* = 0.24, Figure 2B and *p* = 0.32, Figure 2D, respectively), with a frequency of memory anti-tetanus and anti-HBV IgG secreting B cells >0.1% of total IgG secreting B cells (*p* = 0.14 and p=0.17, respectively, compared to CTRL, Figures 2C,E). A competent response was observed also for IgM-secreting B cells (> 10,000 counts/10^6^ stimulated cells of total IgM-secreting B cells in both groups; *p*=0.99, SSNS vs. CTRL), with a frequency of memory anti-tetanus and anti-HBV IgM secreting B cells >4% of total IgM secreting B cells (*p*=0.83, SSNS vs. CTRL for both vaccine-specific responses).

## Discussion

The current study focuses on SSNS pediatric patients at disease onset, prior to any immunosuppressive treatment, in order to investigate the immune and vaccine competence of SSNS patients without confounding effects exerted by an intense immunosuppression steroids, anti-proliferative agents, calcineurin inhibitors and/or B-cell depleting drugs, usually administered in severe forms of SSNS to avoid recurrence of the disease (1). Many reports already investigated the response to previous and subsequent vaccination in INS children and found a reduction of seroprotection induced by previous immunization and an impaired immunogenicity of vaccines administered following the onset of the disease (15–19). However, most of these studies evaluated the levels of vaccine-specific antibodies of INS patients who were under an intense immunosuppression, which can strongly impact the immune response (10). As reported, high-dose prednisone or steroid-sparing agents administered at time of HBV vaccination impair the antibody response (16, 17). In contrast, patients who were vaccinated before starting immunosuppression partially preserve protective titers of anti-HBV IgG (16). However, anti-HBV and anti-tetanus antibodies induced by previous immunization are strongly reduced by a prolonged and intense immunosuppression and by B-cell depletion in INS children (11, 20). B-cell depleting agents are indeed able to efficiently eliminate the circulating memory B-cell subsets, especially in INS patients who received this treatment at an early age (11). Of note, re-immunization following B-cell depletion (after B-cell reappearance) can be effective in restoring vaccine competence in treated patients (11). Another factor that confounds the evaluation of protective antibodies in INS is the reduction of serum IgG that can be dependent on the leakage of immunoglobulins into the urine during the active phase of disease or on an intrinsic immune dysregulation specific of INS patients (2–7, 9). Accordingly, we observed reduced total and anti-tetanus and anti-HBV IgG titers. To overcome this relevant bias, in parallel to the determination of serum vaccine-specific IgG titers, we quantified the number of vaccine-specific memory B cells by an ELISPOT assay. With this approach, we found that circulating B cells in our cohort were highly effective in responding to polyclonal stimulation by producing a large amount of total IgG and IgM. We also observed a competent vaccine-specific memory B-cell response against previous tetanus and HBV immunization. Our study demonstrates that SSNS patients have a competent immune response and a preserved immune memory to previous vaccination against tetanus and HBV at disease onset, before any immunosuppressive therapy.

The main limitation of this study is the limited number of the enrolled patients at disease onset, due to the rarity of the disorder and to the monocentric nature of this pilot study. However, the selection of SSNS patients at onset, prior to any immunosuppression, was necessary to avoid confounding effects of immunosuppressive therapy. More importantly, the experimental approach to quantify the amount of IgG-secreting memory B-cells permitted to overcome the bias of leaked serum IgG into the urine and to correctly evaluate the immune and vaccine competence of the study cohort.

In conclusion, our study demonstrates that SSNS pediatric patients show a preserved immune and vaccine competence at disease onset, which can be efficiently evaluated by quantifying antigen-specific memory B cell response rather than by measuring serum IgG titers. This approach allows early identification of the impairment of the immune and vaccine competence that a protracted use of different immunosuppressive drugs may determine during disease course. Moreover, it overcomes the bias deriving from urinary leakage of serum protein, given that the amount of memory B cells is not affected by proteinuria. Further investigations are necessary to validate our results in a larger cohort of SSNS patients at disease onset and to identify which immunosuppressive drugs affect the vaccine-specific memory B-cell response.

## Data Availability Statement

The raw data supporting the conclusions of this article will be made available by the authors, without undue reservation.

## Ethics Statement

The studies involving human participants were reviewed and approved by Bambino Gesù Children's Hospital, IRCCS, Ethics Committee. Written informed consent to participate in this study was provided by the participants' legal guardian/next of kin.

## Author Contributions

MC designed the study, performed data analysis, and manuscript preparation. EP performed data experiments, data analysis, and helped with manuscript preparation. FZ helped with collection of study samples, clinical information, and manuscript preparation. FC and CC helped with experiments and data interpretation. RC, FE, and MV helped with study design, data interpretation, and manuscript preparation. All authors approved the final version of the manuscript.

## Conflict of Interest

The authors declare that the research was conducted in the absence of any commercial or financial relationships that could be construed as a potential conflict of interest.
